# Waste to worth: diagnostic accuracy of Xpert MTB/XDR on contaminated liquid cultures to salvage the detection of drug-resistant tuberculosis

**DOI:** 10.1128/jcm.00580-25

**Published:** 2025-07-01

**Authors:** Yonas Ghebrekristos, Erick Auma, Zama Mahlobo, Rouxjeane Venter, Natalie Beylis, Jay Achar, Brigitta Derendinger, Sarishna Singh, Megan Burger, Christoffel Opperman, Robin Warren, Grant Theron

**Affiliations:** 1 DSI-NRF Centre of Excellence for Biomedical Tuberculosis Research and SAMRC Centre for Tuberculosis Research, Division of Molecular Biology and Human Genetics, Faculty of Medicine and Health Sciences, Stellenbosch University, Tygerberg 682198, Cape Town, South Africa; 2 National Health Laboratory Service, Greenpoint Tuberculosis Laboratory, Cape Town, South Africa; 3 Division of Medical Microbiology, Department of Pathology, University of Cape Town 536985 , Cape Town, South Africa; 4 Department of Global Public Health, Karolinska Institutet 27106, Stockholm, Sweden; University of Manitoba, Winnipeg, Manitoba, Canada

**Keywords:** Xpert MTB/XDR, contaminated cultures, drug susceptibility testing, tuberculosis

## Abstract

Mycobacterium Growth Indicator Tube (MGIT) 960 culture is critical for tuberculosis (TB) drug susceptibility testing (DST) but is vulnerable to contamination. We evaluated the accuracy of Xpert MTB/XDR, a molecular DST for isoniazid, fluoroquinolone, amikacin, and ethionamide, on to-be-discarded contaminated growth. Xpert MTB/XDR was applied to acid-fast-bacilli-negative, contaminated cultures from sputum from people with rifampicin-resistant TB when Xpert MTB/XDR on sputum was unsuccessful (not resistant or susceptible for all drugs), either at diagnosis (Cohort A) or during treatment monitoring (Cohort B). Future DSTs within 3 months served as a reference standard. We determined potential care cascade improvements. In Cohort A, 10% (66/650) of people had a contaminated culture; 89% (59/66) of contaminated growths were Xpert MTB/XDR TB-positive. Sensitivity and specificity for isoniazid, fluoroquinolone, amikacin, and ethionamide resistance were 100% (95% confidence interval [CI] 85, 100) and 100% (79, 100); 100% (59, 100) and 100% (89, 100); 100% (16, 100) and 100% (91, 100); and 100% (72, 100) and 96% (78, 100), respectively. In Cohort B, 22% (28/129) of people with a contaminated culture were Xpert MTB/XDR TB-positive. Of these, 57% (16/28), 7% (2/28), and 43% (12/28) were isoniazid-, fluoroquinolone-, and ethionamide-resistant (in two, one, and four people, respectively, this would be the first resistant result). In both cohorts, time-to-DST could improve by a median (IQR) of 22 (12–42) days. Xpert MTB/XDR on contaminated MGIT960 cultures had high sensitivity and specificity for DST. This approach could mitigate culture contamination’s negative effects and improve gaps in the drug-resistant TB diagnostic cascade.

**IMPORTANCE:**

Culture contamination is a common impediment to drug susceptibility testing for tuberculosis, the single biggest infectious cause of death globally. Xpert MTB/XDR is a World Health Organization-recommended rapid molecular test for second-line drug resistance. We evaluated Xpert MTB/XDR on contaminated liquid culture growth that would otherwise be discarded, with the people who provided these specimens potentially lost from care cascades. By applying Xpert MTB/XDR to contaminated growth in a high-volume programmatic laboratory, we found the number of people who had second-line DST improved, as did the number of resistant cases diagnosed and time to diagnosis. Furthermore, DST information was generated in people who otherwise would have had none. This approach can therefore reduce the effect of culture contamination on tuberculosis DST, permitting earlier diagnosis and effective treatment initiation and potentially ultimately contributing to improving clinical outcomes and reducing transmission of drug-resistant TB.

## INTRODUCTION

Tuberculosis (TB) remains a global health crisis, with 8.2 million new cases and 1.3 million deaths in 2023. In 2023, an estimated 400,000 rifampicin-resistant cases occurred, but 22% were never diagnosed (1). Furthermore, drug-resistant diagnoses fell by 15% between 2019 and 2022 due to COVID-19 (1). Drug-resistant TB diagnosis requires improvement.

In addition to the better upfront diagnosis of first-line resistance, a key drug-resistant TB care cascade gap is the many people with first-line resistance who do not get second-line drug susceptibility testing (DST). In South Africa in 2023, 12% of multidrug-resistant/rifampicin-resistant (MDR/RR) TB cases did not have second-line DST attempted (1). Furthermore, in those with it attempted, second-line DST was successful (a resistant or susceptible result generated for a second-line drug) in only 66% of people. Due to these unsuccessful results, which can be frequent from paucibacillary sputum, national algorithms often culture sputum in parallel to direct molecular testing (the isolate may be used for repeat molecular testing and/or phenotypic DST). During culture, contamination can occur, and even if people do return to provide another specimen, care cascade delays and loss occur.

These missed opportunities for second-line DST due to contamination are especially important, given the advent of new and repurposed lifesaving Group A drugs (2). Although rapid tests for resistance to these drugs are, for the most part, not yet widely available, resistance is more likely to emerge if the number of effective drugs is low (3). Maximizing second-line DST, including ensuring people with contaminated culture receive appropriate DST as early as possible, is therefore important to protect Group A drugs.

Xpert MTB/XDR (Cepheid, Sunnyvale, USA) is a cartridge-based real-time PCR test endorsed by the World Health Organization in 2021 as a low-complexity reflex test for second-line DST in people with MDR/RR-TB (4). Xpert MTB/XDR must detect *Mycobacterium tuberculosis* complex (MTBC) DNA before it can evaluate whether variants associated with resistance to isoniazid, ethionamide, fluoroquinolone, and second-line injectable drugs (SLIDs), such as kanamycin, amikacin, and capreomycin, are present. The results are automatically interpreted using GeneXpert software (5).

Xpert MTB/XDR is validated on sputum (raw and processed) and culture isolates (6). It has not been evaluated on contaminated Mycobacterium Growth Indicator Tube (MGIT960, Becton Dickinson Systems, USA) cultures. Contaminated cultures, which occur typically at 5%–8% in South Africa, are routinely not further processed and are discarded, prompting a request for a repeat specimen submission for culture (7). However, high rates of repeat specimen non-submission (49%) have been reported in South Africa (7). Furthermore, the median turnaround time (TAT) for sputum resubmission, if it occurs, is 42 days. Finally, it is possible that people who may stand to benefit the most from second-line DST are at elevated risk of culture contamination (because of, for example, microbiome perturbations due to prior antibiotic use from failing regimens, less fit strains caused by drug resistance are more prone to overgrowth by contaminants, especially in clinics in underserved high-burden communities that are less able to adhere to good specimen collection practices).

We have shown promising accuracy results (high sensitivity and specificity) when applying the Xpert MTB/RIF Ultra (Ultra, Cepheid, Sunnyvale, USA) on acid-fast bacilli (AFB)-negative contaminated cultures for the detection of TB and rifampicin resistance (7). The current study aims to evaluate diagnostic performance, with agreement measured against future DST results, and the potential effect on TAT of Xpert MTB/XDR on contaminated MGIT960 growth from people with Xpert MTB/RIF Ultra-diagnosed MDR/RR-TB for drug resistance.

## MATERIALS AND METHODS

### Study design and setting

**Fig 1:**
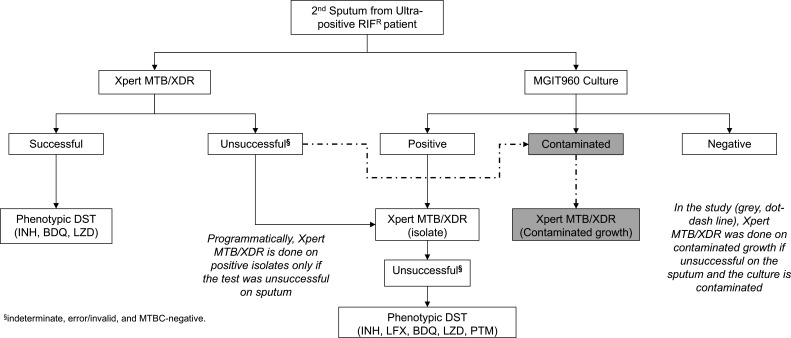
A routine DST algorithm with Xpert MTB/XDR on contaminated cultures added as done in the study (gray, dot-dash line). Abbreviations: BDQ, bedaquiline; DST, drug susceptibility testing; INH, isoniazid, LFX, levofloxacin; LZD, linezolid; MGIT960, Mycobacterium growth indicator tube 960; MTBC, *mycobacterium tuberculosis* complex; PTM, pretomanid; TB-NAAT, tuberculosis nucleic-acid amplification testing.

*Routine specimen processing and programmatic MGIT960 culture:* In this retrospective diagnostic accuracy study, we included people who submitted sputa to the National Health Laboratory Service (NHLS) Greenpoint, Cape Town, South Africa. MGIT960 culture from diagnostic sputum is performed when the Ultra (the initial diagnostic test for individuals with presumptive TB) rifampicin susceptibility result is resistant or indeterminate or for monitoring treatment response (Fig. 1; Supplementary Methods). For initial diagnosis, sputum from people with MDR/RR-TB is used for Xpert MTB/XDR testing in parallel to culture. If Xpert MTB/XDR is unsuccessful, it is re-attempted on the isolate in people who have AFB-positive growth. For treatment monitoring, Xpert MTB/XDR is not done on sputum and only on AFB-positive growth.

**Fig 2:**
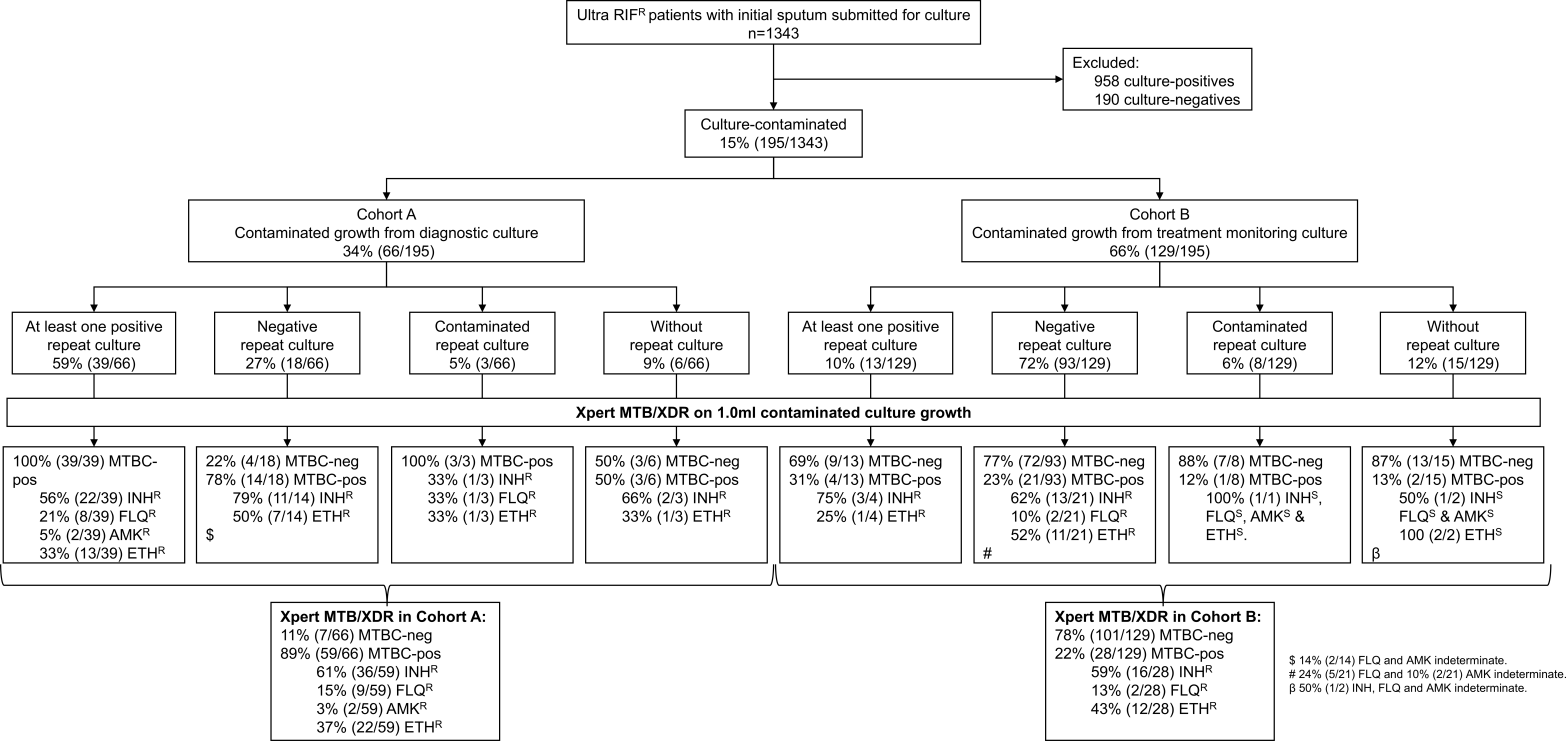
Study profile. We quantified cultures done on Xpert Ultra rifampicin-resistant respiratory specimens in a high-volume programmatic laboratory. Our approach revealed resistance in 52 people for isoniazid, 11 for fluoroquinolone, two for amikacin, and 34 for ethionamide which was programmatically delayed or missed. Xpert MTB/XDR, when done on AFB-negative contaminated growth, is concordant with culture and susceptibility results from a repeat specimen. Abbreviations: AMK^R^, amikacin-resistant; ETH^R^, ethionamide-resistant; FLQ^R^, fluoroquinolones-resistant; IND, indeterminate; INH^R^, isoniazid-resistant; MTBC, *mycobacterium tuberculosis* complex; Ultra, Xpert MTB/RIF Ultra.

*Sample and contaminated culture selection:* MGIT960 growth is automatically monitored, and after a tube is flagged as growth-positive, an acid-fast stain is done. If AFBs are observed, an antigen or molecular test is done to confirm MTBC or non-tuberculous mycobacteria (NTM). If growth occurs but no AFBs are observed, that specimen is reported as culture-contaminated. Contaminated AFB-negative cultures from people with MDR/RR-TB were consecutively collected from 01 October 2023 to 31 March 2024, with samples stored at 2°C–8°C. AFB-positive cultures, including those NTM-positive, were not evaluated. Contaminated cultures were either submitted for diagnosis (from Cohort A; culture done as Xpert MTB/XDR on sputum unsuccessful) or treatment monitoring (Cohort B; culture done to check for positivity and, if positive, resistance). The Cohorts were mutually exclusive. The results of routine TB investigations on specimens or isolates up to 3 months after initial contamination detection were captured (Ultra, culture, Xpert MTB/XDR, phenotypic DST [pDST]). Eligible people were further categorized based on their repeat culture results into four subsets: repeat-culture positives, repeat-culture negatives, repeat-culture contaminated, and those without any repeat culture attempted (Fig. 2).

### Procedures

*Xpert MTB/XDR:* Briefly, 2.0 mL of sample reagent (Cepheid, Sunnyvale, CA, USA) was added to 1.0 mL of contaminated culture, and the sample was processed per the manufacturer’s instructions (6).

*Reference standard:* For TB, people were classified as definite TB if at least one subsequent culture was TB-positive with MTBC confirmed by Ultra on the growth, from a sample within the 3-month follow-up period. If a person only had MTBC-negative repeat results, they were designated TB-negative. For DST, resistant cases had definite TB and resistance on at least one subsequent specimen or isolate within the follow-up period detected using Xpert MTB/XDR and, for isoniazid, phenotypic DST using the 1% indirect proportion method and the MGIT960 liquid culture system (0.1 µg/mL critical concentration). Reference standard susceptible patients had only susceptible results.

### Statistical analysis and definitions

Xpert MTB/XDR sensitivity and specificity for TB and DST were estimated using 2 × 2 tables, with 95% confidence intervals (CIs) calculated via the exact binomial method and Stata (version 18, StataCorp). Diagnostic delay from culture contamination was defined as the days between the initial contamination report date and the earliest subsequent result for TB or DST from a repeat specimen. The date of the earliest subsequent culture-negative result was used for patients with repeat specimens that yielded no positive results. Potential TAT improvements were calculated by comparing diagnostic delay with if Xpert MTB/XDR was done on contaminated growth the day after contamination detection.

## RESULTS

### Quantity of routine diagnostic cultures

In total, 1,343 people had ultra rifampicin-resistant sputum; 48% (650/1343) in Cohort A and 52% (693/1343) in Cohort B. Overall, 71% (958/1,343) were culture-positive, 14% (190/1,343) culture-negative, and 15% (195/1,343) culture-contaminated (66 Cohort A and 129 Cohort B) (Fig. 2). The median age was 37 years (IQR: 30–45), 65% (126/195) of people were men, 65% living with HIV (108/165; 30 unknown HIV status), and 49% had prior TB (94/191; four unknown prior TB status).

### Cohort A (contaminated cultures at initial diagnosis)

In total, 9% (6/66) of people had no repeat specimens. Of those with repeats, 65% (39/60) had at least one repeat specimen culture-positive, 30% (18/60) had all repeat specimen(s) culture-negative (78% [14/18] were Xpert MTB/XDR TB-positive), and 5% (3/60) had only culture-contaminated results for repeats. For TB detection, contaminated growth from 57 (39 plus 18) people hence existed with reference standard information and, for DST, 39 people. Twenty-seven (6 plus 18 plus 3) people had no further DST information.

### Cohort B (contaminated cultures during treatment monitoring)

In total, 12% (15/129) of people had no repeat specimens. Of those with repeats, 11% (13/114) had at least one repeat specimen culture-positive, 82% (93/114) had all repeat specimen(s) culture-negative, and 7% (8/114) had only culture-contaminated repeat result(s). For TB detection, contaminated growth from 106 (13 plus 93) people hence existed with reference standard information and, for DST, three people, and 126 (15 plus 93 plus 8 plus 10) people have no further DST information.

### Xpert MTB/XDR diagnostic accuracy on contaminated cultures

**Fig 3:**
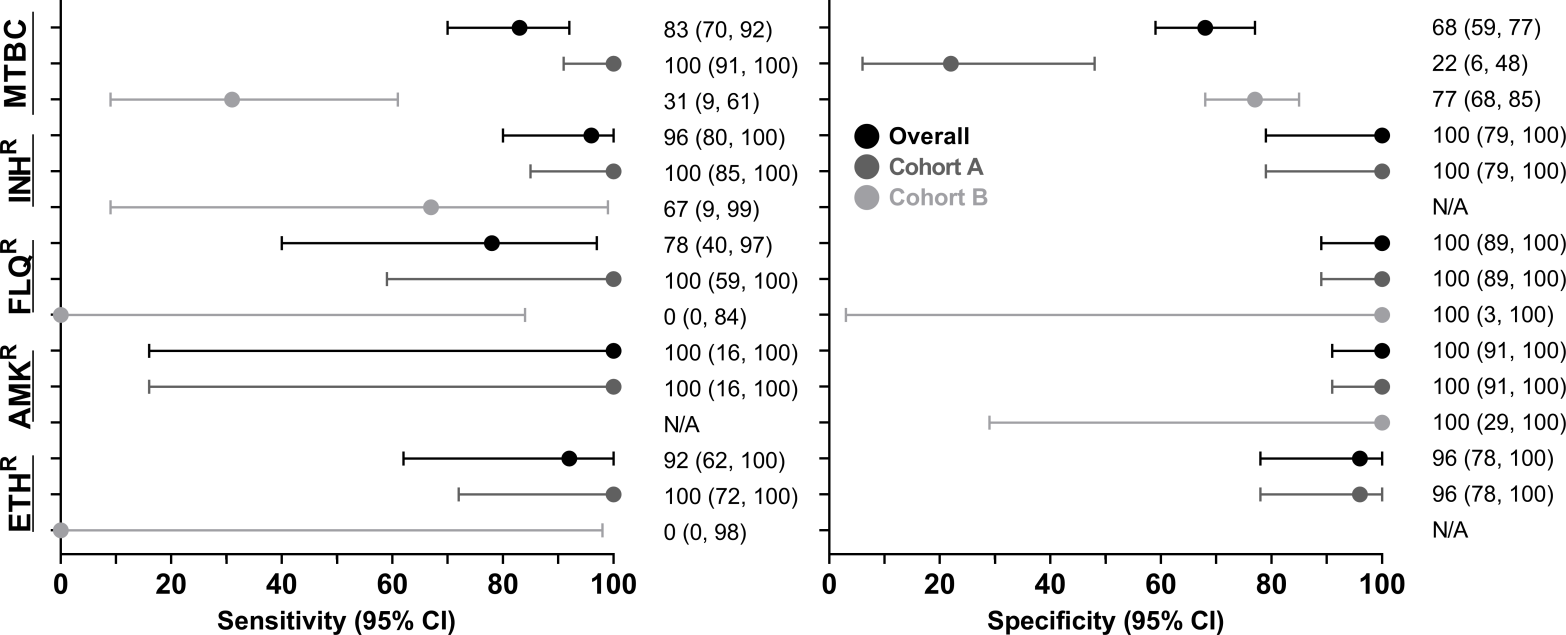
Forest plot of sensitivity and specificity (with 95% confidence intervals) for Xpert MTB/XDR on contaminated cultures. Data are overall and per cohort. As expected, MTBC sensitivity in people on treatment was less than in diagnostic samples (Cohorts B vs. A). Sensitivity for resistance is like that reported for Xpert MTB/XDR on sputum, and specificity is generally excellent, suggesting a Xpert MTB/XDR resistance result from a contaminated culture can be trusted. Cohort B has limited reference standard data for FLQ, AMK, and ETH. The number of people used to calculate each estimate is in Table S1. Abbreviations: AMK^R^, amikacin-resistant; CI, confidence interval; ETH^R^, ethionamide-resistant; FLQ^R^, fluoroquinolones-resistant; INH^R^, isoniazid-resistant; MTBC, *mycobacterium tuberculosis* complex.

*MTBC-detection:* Of 163 people (57 Cohort A, 106 Cohort B) with reference standard information, 48% (78/163) were TB-positive and 52% (85/163) MTBC-negative by Xpert MTB/XDR on contaminated growth. Sensitivity and specificity were 83% (95% CI: 70, 92) and 68% (59, 77), respectively (Fig. 3). In Cohort A, sensitivity and specificity were 100% (91, 100) and 22% (6, 48), and in Cohort B, 31% (9, 61) and 77% (68, 85), respectively. Aggregate data (both Cohorts) for MTBC and DST are on Supplementary page 3.

*Isoniazid susceptibility:* Forty-two people had reference standard information (39 Cohort A and 3 Cohort B). Sensitivity and specificity were 96% (80, 100) and 100% (79, 100), respectively. In Cohort A, sensitivity was 100% (85, 100) and specificity 100% (79, 100). Too few Cohort B people had reference standard information to calculate sensitivity and specificity (all three reference standard resistant), but two were detected as resistant and one false-susceptible by contaminated growth Xpert MTB/XDR.

*Fluoroquinolones susceptibility:* Forty-one people had reference standard information (38 Cohort A and 3 Cohort B). Sensitivity and specificity were 78% (40, 97) and 100% (89, 100), respectively. In Cohort A, sensitivity was 100% (59, 100), and specificity was 100% (89, 100). In Cohort B, two reference standard-resistant people were Xpert MTB/XDR false-susceptible, and one was correctly detected as susceptible.

*Amikacin susceptibility:* Forty-two people had reference standard information (39 Cohort A and 3 Cohort B). Sensitivity and specificity were 100% (16, 100) and 100% (91, 100), respectively. In Cohort A, sensitivity was 100% (16, 100), and specificity was 100% (91, 100). In Cohort B, all three were reference standard susceptible and correctly detected.

*Ethionamide susceptibility:* Thirty-five people had reference standard information (34 Cohort A and 1 Cohort B). Sensitivity and specificity were 92% (62, 100) and 96% (78, 100), respectively. In Cohort A, sensitivity was 100% (72, 100), and specificity 96% (78, 100). In Cohort B, the reference standard resistance person was Xpert MTB/XDR false-susceptible.

### Xpert MTB/XDR results in people without reference standard results

*Cohort A:* Of the 14% (9/66) that did not have reference standard results (six without repeat submission and three repeat culture-contaminated), 67% (6/9) were Xpert MTB/XDR TB-positive, and three of these had resistance to at least one drug detected by Xpert MTB/XDR on contaminated growth (*n* = 1 isoniazid-resistant; *n* = 1 isoniazid, fluoroquinolone, and ethionamide resistant; *n* = 1 isoniazid and ethionamide resistant).

*Cohort B:* Of the 18% (23/129) that did not have reference standard results, 13% (3/23) were TB-positive. Of these, two had no Xpert MTB/XDR-detected resistance on contaminated growth, and one was ethionamide-susceptible and isoniazid-, fluoroquinolone-, amikacin-indeterminate.

### Incremental yield in resistance detection if contaminated Xpert MTB/XDR approach used

In the study period, 90% (1148/1343) of people had a routine Xpert MTB/XDR on sputum or isolate that successfully generated a resistant or susceptible result to at least one drug: 1,141, 1,132, 1,132, and 1,147 for isoniazid, fluoroquinolones, amikacin, and ethionamide, respectively (after removal of indeterminates). For each drug, 522, 93, 45, and 335, respectively, were Xpert MTB/XDR-resistant. Among the 195 people with contaminated culture growth, Xpert MTB/XDR on this growth successfully generated a resistant or susceptible result in 86, 79, 82, and 87 people for each drug, respectively (52, 11, 2, and 34 resistant). The people who successfully received DST therefore increased by 8% (6, 9), 7% (6, 9), 7% (6, 9), and 8% (6, 9), respectively, and resistance diagnosed in the study period would, should the contaminated Xpert MTB/XDR approach be used, have increased by 10% (8, 13), 12% (6, 20), 4% (1, 15), and 10% (7, 14) for isoniazid, fluoroquinolones, amikacin, and ethionamide, respectively (Table 1). In Cohort A, all this additionally diagnosed resistance was resistance hitherto unknown to the program.

**TABLE 1:** Incremental yield^*a*^

Drugs	Number programmatically detected with Xpert MTB/XDR as having resistance (of 1148 tested)	Number in whom Xpert MTB/XDR could not be done programmatically because it was unsuccessful on sputum, culture was contaminated, and Xpert MTB/XDR detected resistance on the contaminated growth (of the 195 tested)	Proportion increases in Xpert MTB/XDR-detected resistance % (95% CI)
INH	522	52	10 (8, 13)
FLQ	93	11	12 (6, 20)
AMK	45	2	4 (1, 15)
ETH	335	34	10 (7, 14)

^*a*^ Number tested by Xpert MTB/XDR programmatically in the study period (on sputum and/or isolates), the number of people in that period in whom this Xpert MTB/XDR could not be done due to contamination, and yield increase in people who had a Xpert MTB/XDR result when the contaminated Xpert MTB/XDR approach was used (and the resulting increase in resistance diagnoses). Abbreviations: AMK, amikacin; CI, confidence interval; ETH, ethionamide; FLQ, fluoroquinolones; INH, isoniazid.

### Cohort B resistance was documented for the first time

Of the 22% (28/129) people Xpert MTB/XDR TB-positive, 21% (6/28) had resistance to a specific drug detected for the first time using the contaminated Xpert MTB/XDR approach. Of these, one had newly diagnosed isoniazid and ethionamide resistance, one newly diagnosed isoniazid resistance, one newly diagnosed fluoroquinolone resistance, and three newly diagnosed ethionamide resistance (these three, which were also isoniazid-resistant, had earlier documented isoniazid resistance).

### Potential turnaround time improvements

The overall potential improved TAT for DST was a median (IQR) of 22 (12–42) days in Cohort A and in Cohort B 28 (12–86) days.

## DISCUSSION

We describe the feasibility of Xpert MTB/XDR on contaminated cultures. Key findings are, compared with previous reports of Xpert MTB/XDR done on sputum (5): (i) sensitivity to detect TB (100% in Cohort A), required for Xpert MTB/XDR to generate DST results, is similar, as is (ii) sensitivity for isoniazid, fluoroquinolone, amikacin, and ethionamide resistance. (iii) Our approach could increase people diagnosed with resistance (12% for fluoroquinolones in our setting), reduce delays and costs associated with the need to collect and culture a second specimen, and generate a DST result in patients who had none before (importantly, many patients had no repeat specimens, meaning the contaminated Xpert MTB/XDR approach would be the only resistant result). Finally, (iv) our approach identified a high proportion of rifampicin-resistant people who were isoniazid-susceptible who, in the event of contamination, could be deprived of or experience delayed initiation of an isoniazid-containing second-line regimen. Together, these findings have implications for reducing laboratory care cascade loss.

We performed Xpert MTB/XDR on contaminated culture growth. This had a similar sensitivity for TB as that reported on sputum (5, 8). All people whose later culture was positive were contaminated gr withowth Xpert MTB/XDR TB-positive. This is important because, if Xpert MTB/XDR had reduced sensitivity for TB on contaminated growth compared with sputum, fewer DST results would be reported due to a failure to detect MTBC.

Xpert MTB/XDR has suboptimal specificity for TB (78% of repeat culture-negative people were Xpert MTB/XDR TB-positive on contaminated cultures), but this false positivity is not clinically consequential because these people are already known to have TB. Xpert MTB/XDR is primarily not intended to be a confirmatory test for TB but rather a reflex DST. This PCR-culture discordance is expected due to time on treatment (during which culturability decreases quicker than DNA positivity (9). The detection of contaminated culture resistance by Xpert MTB/XDR could, even in people whose next culture result is negative, indicate early detection of resistance (10, 11), especially if this resistance was previously not detected.

Sensitivities and specificities for isoniazid, fluoroquinolone, amikacin, and ethionamide susceptibility on contaminated cultures were comparable with those on sputa and isolates (5), with 100% sensitivity for all drugs except fluoroquinolone (one discordant result). The discordant fluoroquinolone result was contaminated culture Xpert MTB/XDR susceptible, but the reference standard was resistant on an isolate collected 87 days later than the sputum that was initially culture contaminated. This may be because of treatment, leading to a selection of an initially hetero-resistant sub-population (10, 11).

Adding Xpert MTB/XDR on contaminated cultures to diagnostic algorithms would lead to more people receiving a molecular test for second-line resistance and a higher diagnostic yield. Importantly, although in our setting, a minority of people eligible for Xpert MTB/XDR on the sputum or isolate did not programmatically receive a Xpert MTB/XDR-resistant or -susceptible result due to culture contamination, we show that if our approach were applied, 4%–12% more resistance (drug dependent) would be identified. This would be associated with improved DST and is most beneficial in people where a repeat specimen was not submitted and who never received second-line DST. Such DST improvements can help TB programs meet targets to find undiagnosed resistance, a WHO EndTB priority (12). This is especially applicable to settings that, unlike ours, have contamination rates more than the “acceptable range” of 3-8% (13) (15%–30% has been reported [7]) and therefore experience higher dropout or delays of people eligible for Xpert MTB/XDR testing on culture isolates. Such environments would benefit the most from this approach.

In addition to detecting second-line drug resistance, our approach confirmed a surprisingly high rate of isoniazid susceptibility (40%) in people already diagnosed as rifampicin-resistant. In South Africa, rifampicin-resistant people are typically given a second-line regimen without isoniazid, whereas isoniazid pDST is done on a culture isolate. However, if their culture was contaminated, such people would be deprived of the rapid inclusion of isoniazid in their regimen. This finding supports other studies challenging the assumption that rifampicin resistance indicates MDR-TB (14, 15), highlighting the potential benefit of Xpert MTB/XDR, and suggests our approach could be used to establish if isoniazid should be included in the regimens of rifampicin-resistant with contaminated cultures.

The results should be interpreted in the context of limitations. Xpert MTB/XDR was done on a different specimen than the reference standard, leading to a possible discordance due to varying bacilli concentration and effects of treatment on strains, but we operated under the assumptions that a Xpert MTB/XDR DST result was better than none and that, should a second specimen be forthcoming, Xpert MTB/XDR retesting of sputum or a culture-positive isolate could always be re-attempted. Our study was also pragmatic, and some people had missing or limited DST data from the lack of follow-up cultures; however, this highlights the need for innovative approaches to addressing gaps in the routine diagnostic pathways (16, 17). Phenotypic DST for all drugs would increase confidence in sensitivity and specificity estimates; however, the study was designed to compare the contaminated culture Xpert MTB/XDR result against other Xpert MTB/XDR results for that patient (on another specimen or culture-positive isolate). Furthermore, isolates were unfortunately not stored in a manner to preserve culturability, meaning retrospective complete pDST was not possible. Laboratories adopting our approach would need to factor in the cost of additional Xpert MTB/XDR testing; however, this is likely offset by the potential reduction in DST of repeat specimens and, most importantly, the long-term benefits associated with more receiving second-line DST.

In conclusion, Xpert MTB/XDR on contaminated cultures is accurate in diagnosing TB and isoniazid, fluoroquinolone, amikacin, and ethionamide resistance. Our approach allows for reduced cascade loss and improved DST TAT. Diagnostic algorithms may consider implementing this approach in laboratories that experience contamination when attempting DST beyond rifampicin resistance.

## Data Availability

Study data can be accessed on request from the corresponding author without restriction.

## SUPPLEMENTAL MATERIAL

Supplemental materials jcm.00580-25-s0001.docx Supplemental methods and results, and Table S1.

ASM does not own the copyrights to Supplemental Material that may be linked to, or accessed through, an article. The authors have granted ASM a non-exclusive, world-wide license to publish the Supplemental Material files. Please contact the corresponding author directly for reuse.
