# Supplementary material for: Waste to worth: diagnostic accuracy of Xpert MTB/XDR on contaminated liquid cultures to salvage the detection of drug-resistant tuberculosis
Source: J Clin Microbiol. 2025 Jul 1;63(8):e00580-25. doi: 10.1128/jcm.00580-25 (PMC12345176; doi:10.1128/jcm.00580-25)
Supplement: Supplemental materials — Supplemental methods and results, and Table S1. [file jcm.00580-25-s0001.docx]

# Supplementary Table of Contents:

[Supplementary Methods 2](#_Toc194935460)

[Routine testing 2](#_Toc194935461)

[Supplementary Results 3](#_Toc194935462)

[Xpert MTB/XDR performance on contaminated cultures 3](#_Toc194935463)

[Supplementary Table 1: 4](#_Toc194935464)

[References 5](#_Toc194935465)

# Supplementary Methods

## Routine testing

Specimens for culture were processed using the standard N-acetyl-L-cysteine (NALC)–sodium hydroxide (NaOH) decontamination method (final NaOH concentration 1.25% in 1.5 ml^1^). Following decontamination, 1.0 ml of processed sediment is aliquoted off, has 2 ml sample reagent added and used for Xpert MTB/XDR per the manufacturer’s protocol^2^. 0.5 ml of the remaining processed specimen was inoculated into a MGIT960 tube as previously described and incubated for ≤42 days^1^. When the instrument automatically flagged tubes as growth-positive, Ziehl-Neelsen microscopy was performed to detect acid-fast bacilli (AFB)^1^. If AFB were observed, MTBC confirmation and RIF resistance were tested using Ultra. If a positive MTBC and RIF resistance result was detected, Xpert XDR was performed on the culture isolate (if direct testing on sediment was not done or unsuccessful) according to the manufacturer’s instructions (**Figure 1**). If no AFBs were observed, growth were classified as "culture contaminated" with no further results provided, in accordance with established programmatic guidelines^3^.

# Supplementary Results

## Xpert MTB/XDR performance on contaminated cultures

*TB-identification results overall:* A total of 195 contaminated cultures (Cohorts A and B) were tested, of which 87 (45%) were TB-positive and 108 (55%) were TB-negative.

*Indeterminate proportion:* An indeterminate proportion of 1% (1/87) for INH, 9% (8/87) for FLQ, and 6% (5/87) for AMK were observed.

*Resistance proportion:* 60% (52/86) were INH-, 14% (11/79) FLQ-, 2% (2/82) AMK-, and 39% (34/87) ETH-resistant.

Cohort A results: 89% (59/66) were TB-positive. Among these, resistance was detected in 61% (36/59) for INH, 16% (9/57) FLQ, 4% (2/57) for AMK, and 37% (22/59) for ETH.

Cohort B results: 22% (28/129) were MTBC-positive. Within this subset, 59% (16/27) were INH-resistant, 13% (2/16) FLQ-resistant, and 43% (12/28) ETH-resistant. In two (INH), one (FLQ) and four (ETH) of these people, resistance was not yet documented.

|  | **N** | **IND** | **TP** | **FN** | **TN** | **FP** |
| --- | --- | --- | --- | --- | --- | --- |
| MTBC  Overall  Cohort A  Cohort B | 163  57  106 | 0  0  0 | 43  39  4 | 9  0  9 | 76  4  72 | 35  14  21 |
| Isoniazid  Overall  Cohort A  Cohort B | 43  39  4 | 1  0  1 | 25  23  2 | 1  0  1 | 16  16  0 | 0  0  0 |
| Fluoroquinolone  Overall  Cohort A  Cohort B | 49  40  9 | 8  2  6 | 7  7  0 | 2  0  2 | 32  31  1 | 0  0  0 |
| Amikacin  Overall  Cohort A  Cohort B | 47  41  6 | 5  2  3 | 2  2  0 | 0  0  0 | 40  37  3 | 0  0  0 |
| Ethionamide  Overall  Cohort A  Cohort B | 35  34  1 | 0  0  0 | 11  11  0 | 1  0  1 | 22  22  0 | 1  1  0 |

Supplementary Table 1: Performance of Xpert MTB/XDR on contaminated culture growth. Data are given overall and for each Cohort. This data is used to produce the forest plot **Figure 3** informing that Xpert MTB/XDR on contaminated culture growth can be trusted.

Abbreviations: FN, false negative; FP, false positive; IND, indeterminate; MTBC, Mycobacterium tuberculosis complex; TN, true negative; TP; true positive.

# References

1. Siddiqi SH, Rüsch-Gerdes S. MGIT procedure manual. *Geneva, Switzerland: Foundation for innovative new diagnostics* 2006: 41-51.

2. Cepheid. Xpert® MTB/XDR Brochure CE IVD 3353 English. 2024.

3. Ghebrekristos YT, Beylis N, Centner CM, et al. Xpert MTB/RIF Ultra on contaminated liquid cultures for tuberculosis and rifampicin-resistance detection: a diagnostic accuracy evaluation. *Lancet Microbe* 2023; **4**(10): e822-e9.
